# The Association Between Maternal Age and the Prevalence of Congenital Heart Disease in Newborns from 2016 to 2018 in Single Cardiac Center in Jeddah, Saudi Arabia

**DOI:** 10.7759/cureus.7463

**Published:** 2020-03-29

**Authors:** Sara T Hashim, Raham A Alamri, Roaa Bakraa, Ranad Rawas, Fayssal Farahat, Rahaf Waggass

**Affiliations:** 1 Medicine, King Saud Bin Abdulaziz University for Health Sciences, Jeddah, SAU; 2 Infection Prevention and Control, King Abdulaziz Medical City, Jeddah, SAU; 3 Pediatric Cardiology, King Saud Bin Abdulaziz University for Health Sciences, Jeddah, SAU

**Keywords:** congenital heart disease, maternal age, advanced maternal age, saudi arabia

## Abstract

Congenital heart diseases (CHD) are suggested to be associated with advanced maternal age in different ethnicities and geographical locations. To provide a profound ground for comparison with Saudi Arabian population, in this study, we have assessed the association between maternal age and congenital heart diseases for different age groups during the period from 2016 to 2018 in King Abdul-Aziz Medical City in Jeddah. In this case-control study, we found no evidence relating maternal age to the incidence of CHD. However, an association between maternal age and the type of CHD was found. Mothers who are 35 years old or younger are more likely to have a baby with atrial septal defects, while babies of mothers who are older than 35 presented mostly with ventricular septal defects and patent ductus arteriosus. Therefore, no modification to the local clinical practice, including a referral of patients for fetal echocardiography based on maternal age, is recommended.

## Introduction

Congenital heart diseases (CHD) represent structural abnormalities of the heart present at birth. These abnormalities can occur within the valves, arteries, veins, and walls of the heart. They are the most prevalent type of birth anomalies, occurring in approximately from three to nine for every 1,000 live births, and represent a considerable proportion of infant mortality due to birth defects [1]. Furthermore, studies in different regions of the world suggested that congenital heart diseases can be multifactorial involving genetic and non-genetic risk factors, including co-chromosomal abnormalities, maternal pre-gestational diabetes, maternal hypertension, and infant’s sex [2-4]. Also, maternal age is thought to be a considerable risk factor for congenital heart diseases. The most common congenital heart diseases which are linked with advanced maternal age are coarctation of the aorta and valvular pulmonic stenosis [5]. 

Many studies around the world suggest an association between different maternal age groups and CHD. While some studies have illustrated the U-shaped association, others have reported a linear relationship with the increased strength of association with advanced maternal age [6-7]. For example, research in Hawaii proved the association between advanced maternal age ≥35 and ventricular septal defect, atrial septal defect, endocardial cushion defect, hypoplastic left heart syndrome [7]. However, a study conducted in the United Kingdom reported little evidence of the association between advanced maternal age, ≥35 at delivery, and the incidence of CHD [5]. Comparatively, the literature on maternal age as an isolated significant risk factor of CHD in Saudi Arabia has been limited. The study in Riyadh reported that maternal age plays a significant role in consanguinity in congenital heart disease [8]. However, there is no study performed in Jeddah regarding this matter. All in all, there has been a discrepancy regarding the strength and direction of association in different age groups. 

This study aims to elaborate on the association between maternal age and congenital heart diseases for different age groups based on data gathered from 2016 to 2018 in King Abdul-Aziz Medical City in Jeddah, Saudi Arabia. It is of particular importance to gather our local data to provide a profound understanding of our public health status and a ground for comparison with data from different ethnicities and geographical locations. In addition, depending on the results, the data collected might suggest some modifications to the local clinical practice, including a referral of patients for fetal echocardiography based on maternal age alone. 

## Materials and methods

A case-control study was conducted in King Faisal Cardiac Center in King Abdulaziz Medical City, Jeddah, during the period from January 1, 2016, to December 31, 2018. The study included infants diagnosed with non-chromosomal CHD and their mothers and non-CHD infants and their mothers. Infants with chromosomal CHD, premature or newborn infants less than six weeks of age with patent ductus arteriosus, were excluded. Assuming the likelihood of non-chromosomal CHD among live births of mothers 35 years or older is twice compared to younger age mothers (odds ratio around two) (Miller et al.), then the required sample size is 351 for the cases group and 1,053 for the control group, at 95% confidence interval and 80% study power [1]. All cases with non-chromosomal CHD during the study period were reviewed. When the required sample size was not met, the study was expanded to include more cases. For each case, three consecutive live birth controls (medical records numbers following the identified case) were performed. The controls were identified based on the list of the birth cohorts. A checklist has been used to collect data that included maternal risk factors such as age in years, chronic conditions, consanguinity, medications, and newborn outcomes including gender, diagnosis of CHD, type of CHD.

SPSS software version 24 (IMB Inc., Armonk, USA) and descriptive statistics (frequency, percentage, mean, standard deviation) were applied for data analysis. Chi-square test was used for categorical variables (diagnosis of CHD and maternal age category 35 years or younger vs. older than 35 years). Logistic regression analysis and odds ratio with 95% confidence interval were applied to identify the association of different risk factors on the occurrence of CHD among live births. The level of significance was determined at 0.05.

## Results

This study focused on the incidence of CHD related to maternal age. The data was collected from 1,128 babies and their mothers. 

**Figure 1:**
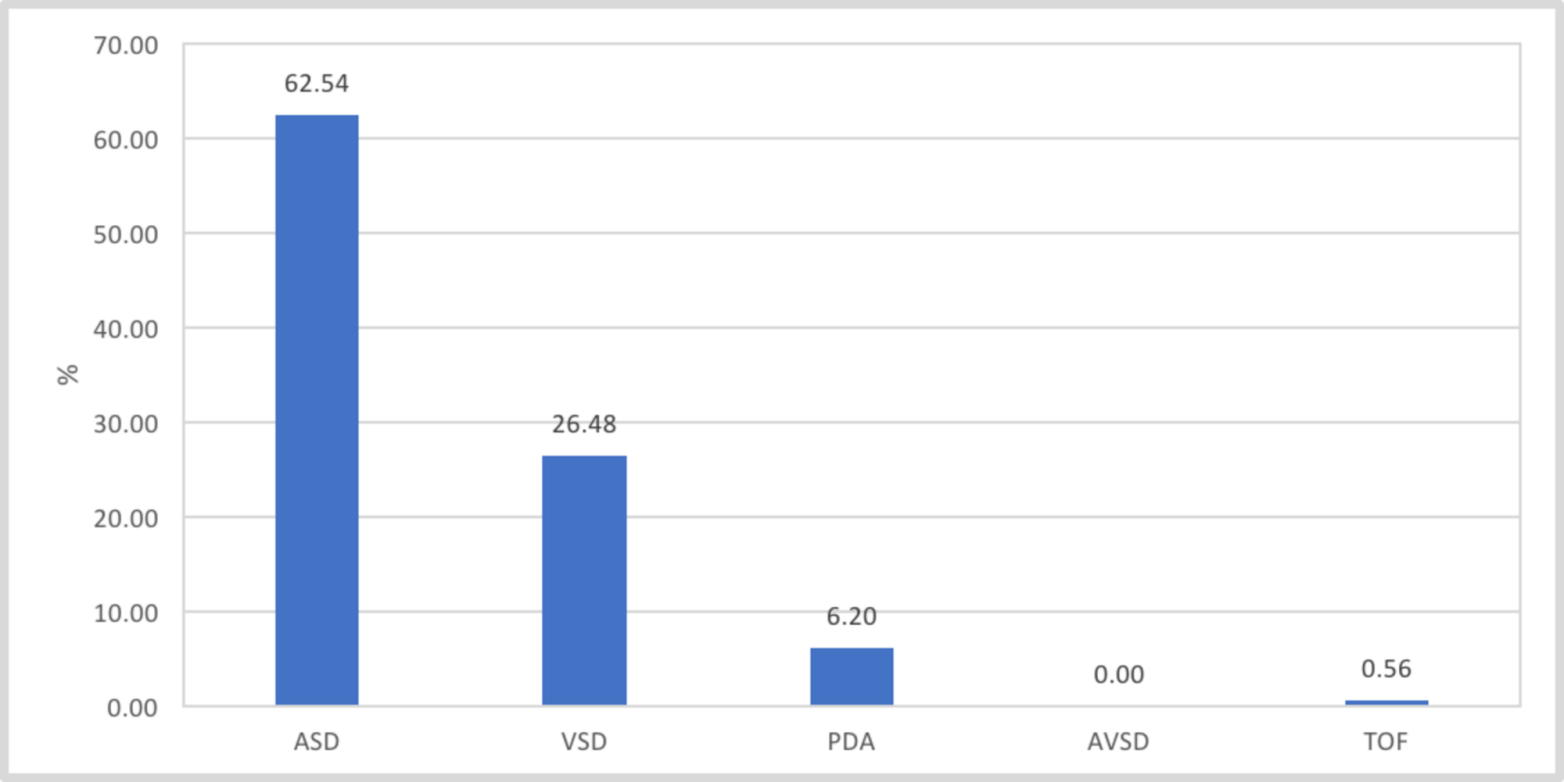
Incidence of different type of congenital heart diseases ASD - atrial septal defects; VSD - ventricular septal defects; PDA - patent ductus arteriosus; AVSD - atrioventricular septal defect; TOF - tetralogy of fallot

**Figure 2:**
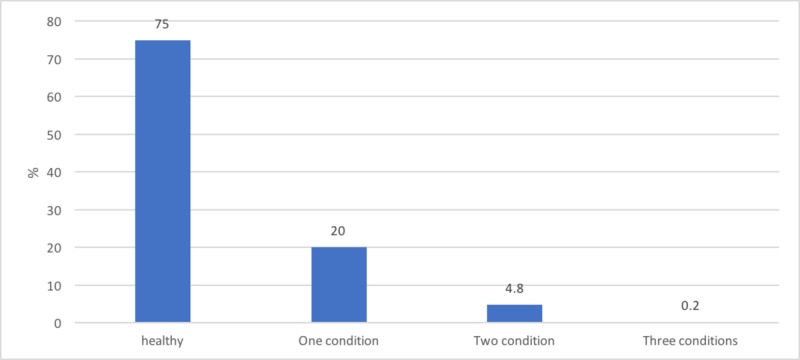
The incidence of multiple conditions

The study found that most CHD babies were diagnosed with atrial septal defects (ASD) followed by ventricular septal defects (VSD) then less frequently with patent ductus arteriosus (PDA). Among 282 newborns with CHD, 222 (62.54%) of them had ASD, 94 (26.48%) had VSD, and 22 (6.20%) had PDA (Figure1). The majority of the patients were diagnosed with one condition. However, there was a small group that had multiple conditions (Figure2). The study included 547 females and 581 males, and it showed no significant relationship between the gender of the baby and the incidence of CHD.

**Figure 3:**
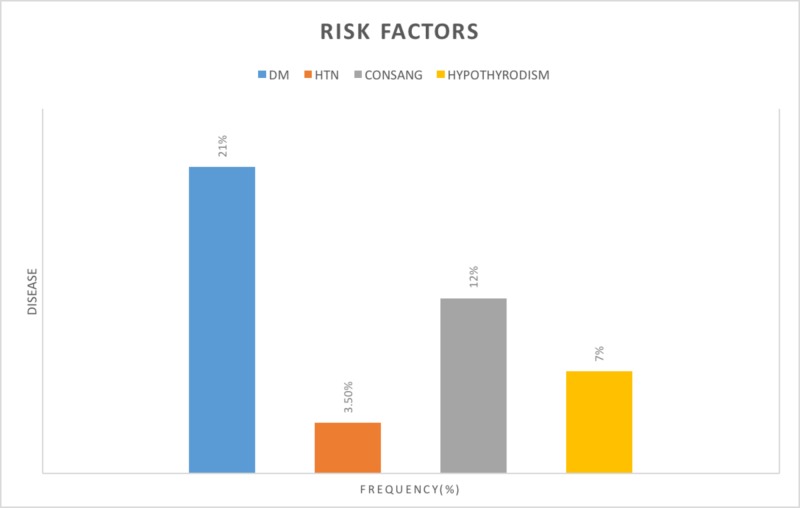
Mothers' risk factors DM - diabetes mellitus; HTN - hypertension; CONSANG - consanguinity

Mothers with CHD babies were found to have risk factors such as diabetes, hypertension, consanguinity, and hypothyroidism (Figure 3). Those factors have been studied in many studies before as contributive factors in the incidence of CHD. 

**Figure 4:**
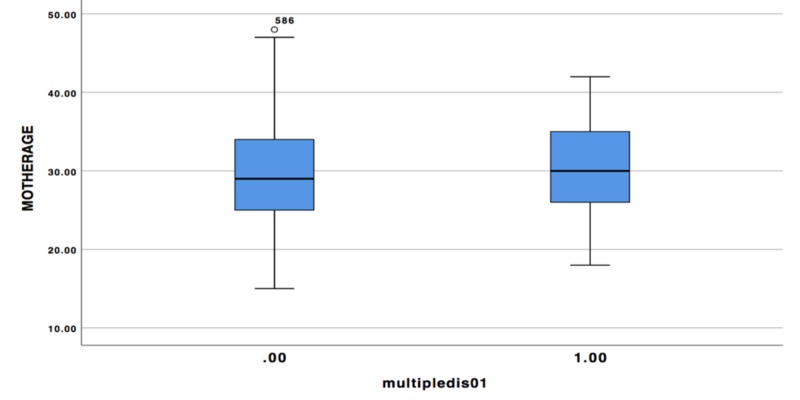
The average ages of both groups of mothers

The study focused on the maternal age as the main risk factor in CHD babies, and it showed that the mean of the age between diseased patients and the control group is almost the same, p=0.182 (Figure4).

## Discussion

In this study, the results showed that the mean age of the mothers with CHD babies and the control group is almost the same, and it showed no statistical differences. However, another important finding is that there was a significant association between maternal age and the type of CHD. The mothers who are older than 35 years are more likely to have babies with VSD and PDA, while mothers aged 35 years and younger are more likely to have babies with ASD (Table1).

**Table 1:** The relation between the types of CHD and mother's age CHD - congenital heart diseases; ASD - atrial septal defects; VSD - ventricular septal defects; PDA - patent ductus arteriosus; AVSD - atrioventricular septal defect; TOF - tetralogy of fallot

Congenital Heart Disease			Mother age ≤ 35	Mother age > 35	P-value
ASD	No	Number (%)	724 (79.1)	182 (85.3)	0.037
	Yes	Number (%)	191 (20.9)	31 (14.6)	
VSD	No	Number (%)	847 (92.6)	187 (87.8)	0.023
	Yes	Number (%)	68 (7.4)	26 (12.2)	
PDA	No	Number (%)	901 (98.5)	205 (96.2)	0.034
	Yes	Number (%)	14 (1.5)	8 (3.8)	
TOF	No	Number (%)	913 (99.8)	213 (100)	0.495
	Yes	Number (%)	2 (0.2)	0	

The differences between this study and the other studies could be due to the differences in maternal age distribution groups. Another difference is the inclusion of chromosomal abnormalities cases and consanguinity in previous studies. The limitations of this study are the small sample size, which gives the study low statistical power of the analyses. Another limitation is that the results have been collected from King Faisal cardiac center in Jeddah only, so they may not apply to the whole of Saudi Arabia. As in the future, it will be helpful to study different regions in Saudi Arabia as the location has a huge impact on the tribe's genetic heritage and diseases. Another limitation is related to data collection from the Excelera® system, which includes all the babies who are suspected of having heart abnormality and referred to echocardiography; it was found that there was a huge number of newborns who are suspected of having CHD per year which required more time to process and study.

## Conclusions

The average age of both maternal age groups was almost the same (less than 35 years). However, an association between maternal age and the type of CHD in infants was made. It was found that mothers who are 35 years old or younger are more likely to have an ASD type new-birth, while mothers who are older than 35 years are having new births with VSD and PDA. Therefore, no modification to the local clinical practice, including a referral of patients for fetal echocardiography based on maternal age, is recommended.
